# Jugular venous catheter-associated thrombosis and fatal pulmonary embolism: A case report

**DOI:** 10.1097/MD.0000000000020873

**Published:** 2020-06-26

**Authors:** Jinrong Wang, Lili Wang, Huimian Shang, Xiaoya Yang, Shufen Guo, Yanling Wang, Chaobo Cui

**Affiliations:** Department of Critical Care Medicine, Harrison International Peace Hospital, Hebei, China.

**Keywords:** bedside emergency ultrasound, catheters related thrombosis, pulmonary embolism

## Abstract

**Introduction:**

Central venous catheters related thrombosis (CRT) insertion has been shown to increase the risk of venous thromboembolism, particularly pulmonary embolism (PE). Nevertheless, deaths cased due to PE have been rarely reported.

**Patient concerns:**

A central venous catheter was introduced through the right jugular vein during the operation due to severe septic shock from a 57-year-old male patient. Two days after surgery, the hemodynamics was stable. On the 7th day, and low molecular weight heparin calcium (4100 units, once a day) was added for anticoagulation to prevent venous thromboembolism. On the 15th day, during the process of central venous catheter removal, the patient suddenly lost consciousness, suffered cardiac arrest, and received emergency cardiopulmonary resuscitation.

**Diagnosis:**

Jugular venous catheter-associated thrombosis and fatal PE. An acute bedside ultrasound showed a thrombus drifting with the blood stream in the right jugular vein. The lower section of the xiphoid process by echocardiography showed decreased systolic amplitude of the right atrium and right ventricle, widened and fixed inferior vena cava, and no variation with respiration. Para-sternal left ventricular long axis section showed that the right ventricular outflow tract was significantly extended, and the contraction amplitude of the anterior and posterior walls of the left ventricle decreased. Left ventricular short axis section indicated a right ventricle enlargement and ventricular septum deviation of left ventricle, showing “D” sign. Apical 4-chamber view showed that the right ventricular ratio increased and the contractile capacity decreased.

**Interventions:**

One and a half million units of urokinase were immediately given trough intravenous drip.

**Outcomes:**

Twenty minutes after thrombolysis, the patient's autonomic heart rhythm was recovered, but continued to suffer from hypotension and coma, followed by multiple organ failure, and died 50 hours later.

**Conclusion:**

Recent clinical practice guidelines recommend against the routine use of any anticoagulant thromboprophylaxis in patients with central venous catheters, but for patients at particularly high risk for CRT, consideration can be given to using higher doses of anticoagulant as prophylaxis, although there are virtually no data to support this approach.

## Abstract 2

Supplemental Digital Content is available in the text

## 1 Introduction

Central venous catheters (CVCs) are one of the most commonly use interventions for critically ill patient. CVCs-related thrombosis (CRT) is the most common noninfectious complication of CVCs insertion. The incidence of CRT is estimated to be 0.4 to 1.0 per 10,000 persons.^[1]^ During anticoagulation therapy, 8% of patients with CRT may present with a concomitant pulmonary embolism (PE).^[2]^ Nevertheless, deaths cased due to PE have been rarely reported. Here we present a single case of a 57-year-old male patient who was diagnosed with jugular venous catheter-associated thrombosis and died of fatal PE.

## 2 Case presentation

**Figure 1:**
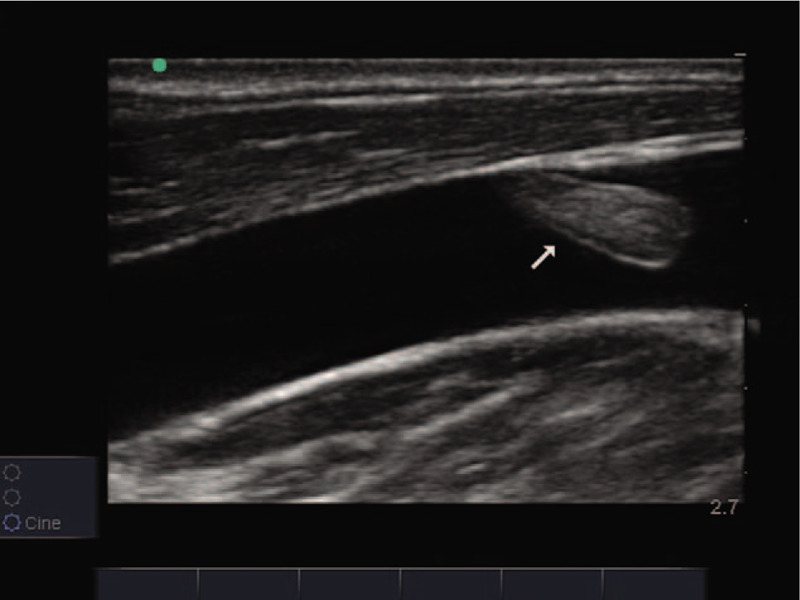
Long axial section of the right jugular vein showing thrombus attached to the inner wall of the vessel (white arrow).

**Figure 2:**
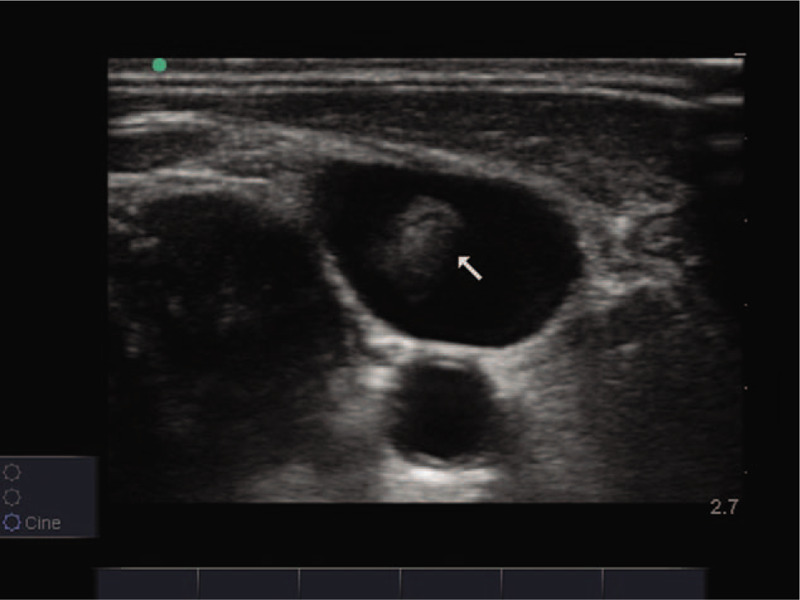
Short axial view of the right jugular vein showing thrombosis (white arrow).

**Figure 3:**
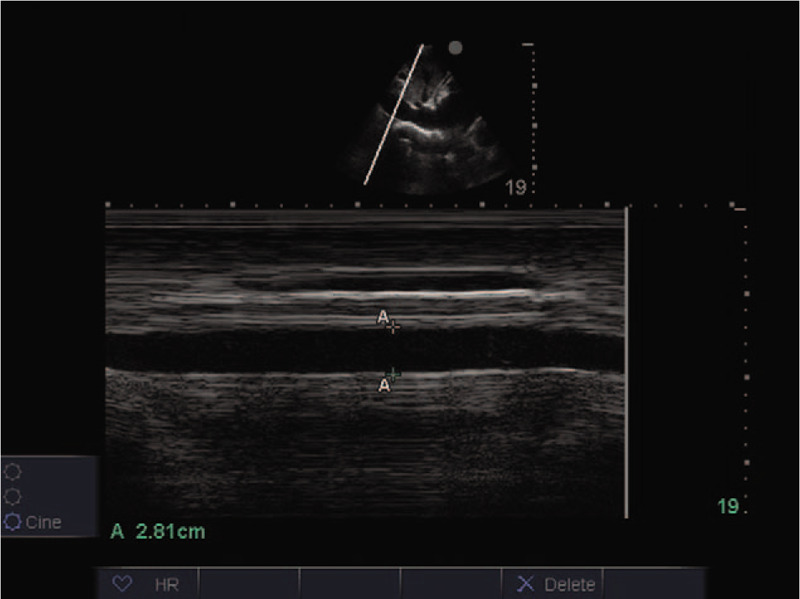
Widened and fixed inferior vena cava without variation with respiration.

A 57-year-old male patient was admitted to the hospital due to acute abdominal pain and diffuse peritonitis that lasted for 3 days. Due to severe septic shock, a central venous catheter was introduced through the right jugular vein during the operation to facilitate rapid fluid resuscitation and the application of vasoactive drugs. Two days after surgery, the hemodynamics was stable. On the third day, the patient had spontaneous exhaust and got out of bed with slight movement. The vasoactive agent was discontinued on day 5. On the 7th day, patient was given a small amount of liquid diet; parenteral nutrition fluid was introduced through central venous catheter, and low molecular weight heparin calcium (4100 units, once a day) was added for anticoagulation to prevent venous thromboembolism. On day 14, the patient was able to eat normally and had to stop parenteral nutrition treatment. On the 15th day, during the process of central venous catheter removal, the patient suddenly lost consciousness, suffered cardiac arrest and received emergency cardiopulmonary resuscitation. An acute bedside ultrasound showed a thrombus drifting with the blood stream in the right jugular vein (Figs. 1 and 2; Additional file 1 [Long axial section of the right jugular vein showing the thrombus attached to the inner wall of the vessel and drifting with the blood stream.] and Additional file 2 [A short axial view of the right jugular vein shows thrombus drifting with blood flow.]). The lower section of the xiphoid process by echocardiography showed decreased systolic amplitude of the right atrium and right ventricle (Additional file 3 [Lower section of the xiphoid process shows decreased systolic amplitude of the right atrium and right ventricle.]), widened and fixed inferior vena cava, and no variation with respiration (Fig. 3). Para-sternal left ventricular long axis section showed that the right ventricular outflow tract was significantly extended, and the contraction amplitude of the anterior and posterior walls of the left ventricle decreased (Additional file 4 (Long axis view of the parasternal left ventricle showed that the right ventricular outflow tract widened significantly and the contraction amplitude of the anterior and posterior walls of the left ventricle decreased.)). Left ventricular short axis section indicated a right ventricle enlargement and ventricular septum deviation of left ventricle, showing “D” sign (Additional file 5 (Short axial section of left ventricle shows right ventricle enlargement and ventricular septum deviation of left ventricle, showing “D” sign.)). Apical 4-chamber view showed that the right ventricular ratio increased and the contractile capacity decreased (Additional file 6 (4-chamber view of apical heart showed increased right ventricular ratio and decreased contractility.)). In consideration of fatal pulmonary embolism, 1500,000 units of urokinase were immediately given trough intravenous drip. After 20 minutes, his autonomic heart rhythm was recovered, but continued to suffer from hypotension and coma, followed by multiple organ failure, and died 50 hours later. Because the patient's condition has been in an extremely dangerous state, the pulmonary embolism was not diagnosed by computed tomography pulmonary arteriography (CTPA).

## 3 Discussion

### 3.1 Pathophysiology

CVCs impact each component of Virchow triad: stasis, hypercoagulability, and endothelial injury. CVCs insertion results in local vessel wall injury, activating the coagulation and proinflammatory cascades. Continuous friction of CVCs against the vessel wall as well as turbulent inflow from the catheter and the toxic effects of some medications promote ongoing endothelial injury and thrombus formation. In addition, the presence of CVCs in the vessel lumen slows blood flow, leading to stasis, and the synthetic materials used to construct CVCs likely activate coagulation^3^.

Thrombus can form within, surrounding, or at the tip of the catheter. If thrombus forms around the catheter, when the catheter is removed, thrombus falls off the catheter and travels through the bloodstream into the pulmonary artery, causing a fatal pulmonary embolism^4^. The internal jugular vein thrombosis detected by ultrasound in this case not only confirmed the formation of CRT, but also indicated that part of the thrombosis had not yet fallen off the internal jugular vein wall.

### 3.2 Risk factors

Concomitant pulmonary embolism occurs less frequently in CRT, but it is associated with a worse outcome. So far, many risk factors for CRT have been reported.^[3]^ Risk factors for CRT can be grouped into device-, patient-, and treatment- related factors. Device-related factors can be divided in the following: multiple insertion attempts,^[4]^ large catheter size to vein diameter ratio,^[5,6]^ catheter infection,^[7]^ improper catheter position (not at atriocaval junction),^[8]^ number of lumens and catheter size (6 F triple-lumen; >5 F double-lumen; >4 F single-lumen),^[9–11]^ and catheter material.^[12]^ CRT risk is associated with the insertion site, with the femoral vein being the highest risk site, followed by the jugular vein and then the subclavian vein.^[8,11,13]^ In addition, patient-related factors may also influence the CRT risk. Older age and body mass index have been associated with higher risk compared with sex and ethnicity.^[14]^ In addition, cancer and critically ill patients are at increased risk with catheter-related infections.^[14–16]^ The presence of inherited thrombophilia as well as a personal history of venous thromboembolism (VTE) increase the risk of CRT.^[8,15,17]^ Treatment related risk factors for CRT include chemotherapy, surgery and parenteral nutrition, whereas therapeutic anticoagulation reduces the risk of thrombosis.^[10,14,18]^ In this case, CRT risk factors were associated with emergency surgery, parenteral nutrition, and severe systemic infection. Although low molecular weight heparin calcium was given during the course of the disease to prevent thrombosis, CRT was not avoided.

### 3.3 Diagnostic strategy

Suspected high-risk PE is a life-threatening situation. The clinical probability is usually high, and the differential diagnosis includes acute valvular dysfunction, tamponade, acute coronary syndrome, and aortic dissection. Bedside transthoracic echocardiography is considered the most common diagnostic tool for this condition that may yield evidence of acute pulmonary hypertension and right ventricle (RV) dysfunction if acute PE is the cause of the patient's hemodynamic decompensation. In a highly unstable patient, echocardiographic evidence of RV dysfunction is sufficient to prompt immediate reperfusion without further testing. This decision may be strengthened by the (rare) visualization of right heart thrombi. As soon as the patient can be stabilized by supportive treatment, final confirmation of the diagnosis by CT angiography should be performed.^[19–22]^ During the removal of the catheter, our patient experienced cardiac arrest. Echocardiography showed enlargement of the right ventricle, deviation of the ventricular septum to the left ventricle, presence of pulmonary hypertension, and thrombosis in the jugular vein. PE can be diagnosed despite failure to undergo CTPA.

### 3.4 Catheter removal

For CRT patients that no longer need central venous access, the 2016 ACCP guidelines recommend catheters removal.^[23]^ Reasons to remove the catheter include concomitant catheter-related infection, failure of symptoms to resolve with anticoagulation alone, or no need for continued vascular access.^[3]^ No specific recommendation is provided regarding the duration of anticoagulation prior to removal to minimize the risk of embolization. Although there are no data that provide guidance, an initial period of anticoagulation (at least 7 days if possible) prior to catheters removal is preferred to prevent thromboembolism and should be considered on a case-by-case basis based on the location and size of the thrombus burden, the risk for embolization and related complications upon catheters removal; as well as the risk of bleeding with anticoagulation and the potential complications resulting from delayed removal.^[24]^

### 3.5 Prevention of CRT

Prevention practices should target patient-, treatment-, and device related risk factors for CRT. For example, clinicians should try to avoid placing CVCs or use the smallest caliber catheter possible, ensure proper catheter tip location, and remove CVCs when they are no longer required. Measures to prevent catheter-related infections can reduce CRT risk.

Anticoagulant thromboprophylaxis has been evaluated in multiple randomized trials of patients with CVC, leading to several recent systematic reviews.^[25–28]^ A meta-analysis of 15 randomized trials found a significant benefit of anticoagulant prophylaxis for both asymptomatic and symptomatic CRT.^[29]^ Nevertheless, a more recent systematic review found that both prophylactic heparin and low dose vitamin K antagonist have a nonsignificant trend toward a decrease in symptomatic DVT.^[25]^ A largest study of thromboprophylaxis in CVC randomized 1590 cancer patients show that symptomatic CRT was less frequent in the patients given adjusted-dose warfarin than in those who received no prophylaxis, but major bleeding was more common.^[30]^ Analyses that pooled trials of prophylactic low molecular weight heparin (LMWH) found a nonsignificant trend toward greater efficacy in preventing asymptomatic deep vein thrombosis (DVT) compared with no prophylaxis without increased bleeding.^[31,32]^ Additionally, a most recent study for patients receiving chemotherapy through a CVC found anticoagulant prophylaxis was associated with fewer asymptomatic DVTs and symptomatic DVTs, but more bleeding, and there were no significant differences between warfarin and LMWH.^[33]^

This patient is at high risk for CRT due to risk factors such as surgery, shock, catheterization, and parenteral nutrition. There was no significant risk of bleeding and anticoagulant prophylaxis could be applied in the early postoperative period. This patient received LMWH prophylaxis 7 days after the operation, which was somewhat late. In addition, although there is no evidence that routine ultrasound screening for CRT is required prior to catheter removal. However, according to clinical experience, ultrasonic screening before catheter removal can detect asymptomatic thrombosis at an early stage, and drug anticoagulant therapy can be given in time to reduce the risk of pulmonary embolism.

## 4 Conclusion

In summary, research suggests that the use of prophylactic doses of LMWH is not very effective in preventing CRT; nevertheless, this approach is safe. Recent clinical practice guidelines recommend against the routine use of any anticoagulant thromboprophylaxis in patients with a CVC.^[26–28,34]^ However, for patients at particularly high risk for CRT, for example, those with previous CRT, consideration can be given to using higher doses of anticoagulant as prophylaxis, although there are virtually no data to support this approach, which may also increase bleeding risk.^[30]^

## Author contributions

**Conceptualization:** Jinrong Wang.

**Data curation:** Jinrong Wang, Lili Wang, Huimian Shang, Chaobo Cui.

**Formal analysis:** Huimian Shang, Shufen Guo, Chaobo Cui.

**Writing – original draft:** Lili Wang, Yanling Wang.

**Writing – review & editing:** Xiaoya Yang.

## Supplementary Material

Supplemental Digital Content

## Supplementary Material

Supplemental Digital Content

## Supplementary Material

Supplemental Digital Content

## Supplementary Material

Supplemental Digital Content

## Supplementary Material

Supplemental Digital Content

## Supplementary Material

Supplemental Digital Content
